# Analyzing generalized coherent states for a free particle

**DOI:** 10.1038/srep30538

**Published:** 2016-08-11

**Authors:** Mustapha Maamache, Abderrezak Khatir, Halim Lakehal, Jeong Ryeol Choi

**Affiliations:** 1 Laboratoire de Physique Quantique et Systèmes Dynamiques, Faculté des Sciences, Université Ferhat Abbas Sétif 1 Sétif 19000, Algeria; 2 Faculté des Sciences Exactes, Université des Frères Mentouri, Constantine, Route d’Ain El Bey, Constantine, Algeria; 3 Department of Radiologic Technology, Daegu Health College, Yeongsong-ro 15, Buk-gu, Daegu 41453, Republic of Korea

## Abstract

Despite the didactic importance of a free particle in quantum mechanics, its coherent state analysis has long been untouched. It is only recently that it has been noticed and studied in the semiclassical domain. While the previously known solutions, reported by Bagrov *et al*. for a free particle, are described using the linear non-Hermitian invariant operator, we show in this work that the general solution of the Schrödinger equation can also be naturally derived using a simpler method based on an Hermitian linear invariant operator. According to this, an exact Gaussian wave function that corresponds to a coherent state solution is obtained. An interpretation for such general quantum solution designated within the Lewis-Riesenfeld framework is provided and, further, quantum-classical correspondence principle for the system is reexamined.

The derivation of the Schrödinger solutions is the most central task when we investigate quantum properties of a specific system. The usual method for obtaining Schrödinger solutions is the separation of variables method that is based on mathematical techniques separating out the time function from the Schrödinger equation. Solving the time-*in*dependent Schrödinger problem leads to derive analytically the wave functions representing the energy eigenstates (and the corresponding eigenvalues) of a given problem. Although this simple method is valid for a free particle, the eigenstates of the corresponding Hamiltonian cannot be normalizable. These states correspond to plane waves which are fully delocalized. In this work, we will study Gaussian-like states describing the semiclassical time behavior of a localized free-particle. For the sake of brevity, we will call them “coherent state of a free particle” since the Gaussian structure of the state is preserved during the time evolution.

However, such unnormalizable solutions can be customarily superimposed to be localized solutions by means of a Fourier transformation. Notice that the localized solutions derived in this way are no longer eigenstates of the Hamiltonian although they satisfy the Schrödinger equation. One of the simplest examples for this procedure is to construct the Gaussian wave packets that correspond to the coherent states which describe the semiclassical motion of a localized particle.

A class of the well known coherent states is those of the simple harmonic oscillator^1^,^2^,^3^,^4^; they were originally obtained by Schrödinger^1^ as specific quantum states, where the expectation values of the position and momentum operators in these states were the same as the corresponding classical solutions. These states have a number of other interesting properties including the followings: (a) They are eigenstates of the destruction operator; (b) They are created from the ground state by a unitary operator; (c) They minimize the uncertainty relations and do not spread over time; (d) They are (over)complete and normalized, but not orthogonal. These properties have been fully demonstrated in the literature^5^,^6^,^7^,^8^,^9^,^10^,^11^. Thus, the definition and properties of the coherent states for the simple harmonic oscillator are well understood in the context of theoretical physics.

However, despite the didactic importance of analyzing quantum characteristics of a free particle as well as that of the harmonic oscillator, coherent states of a free particle have not been extensively investigated because of the difficulties in deriving their explicit analytical forms. It has been shown that the group-theoretic approach to the time evolution of quantum states is equivalent to the corresponding Lewis-Riesenfeld approach and the free-particle dynamics can be reproduced from the dynamics of the time-dependent harmonic oscillator by letting *ω*(*t*) → 0 for *t* → ∞^12^. It is only recently that quantum dynamics of a free particle has been noticed and the time behavior of its semiclassical wave packets has been studied^13^,^14^,^15^,^16^.

In ref. 13, Bagrov *et al*. constructed coherent states for a free particle as Gaussian wave packets that allow one to establish a natural relation between the classical and quantum descriptions of a free particle. They used essentially an invariant operator method by introducing a non-Hermitian time-dependent linear invariant described in terms of momentum *p* and position operators *x*^17^. In the approach based on such a non-Hermiticity assumption (NHA) for the invariant operator, a time-dependent generalized coherent state can be derived by solving the eigenvalue equation of the non-Hermitian linear invariant (or of the non-Hermitian linear integrals of motion) under some constraints on the parameters and constants^13^. For other papers exploiting similar techniques for deriving Schrödinger solutions of specific systems, you can refer to refs 18, 19, 20.

Recall that, according to the theory of Lewis and Riesenfeld^21^, an invariant is an operator that must necessarily satisfy three requirements: (a) It is Hermitian; (b) It satisfies the von Neumann equation; (c) Its eigenvalues are real and time-*in*dependent. Furthermore, any invariant satisfying these three requirements leads to a complete set of solutions of the corresponding Schrödinger equation. So, a conventional solution is constructed as a linear combination of these solutions. In general, any two different Lewis-Riesenfeld Hermitian invariants lead to two different sets of solutions. However, each solution of one of the invariants can be written as a linear combination of the solutions of the second one. Considering the above properties of the Hermitian linear invariant operator, we will show that the Gaussian wave packet and all the results for the coherent states of a free particle can be obtained by using the Hermitian invariant operator which is linear in *p* and *x*. It should be noted that this linear invariant operator can be applied not only for the free particle, but also for other systems that are quadratic in *p* and *x* such as the harmonic oscillator^18^,^19^,^22^.

In the present paper, we propose an alternative and simpler method for deriving Schrödinger solutions for the motion of a free particle on the basis of a Hermiticity assumption (HA) for the linear invariant operator. The merit of the research using HA is that we can derive complete quantum solutions of the system without imposing any conditions on the parameters necessary for defining the invariant; we will show that this method leads to obtaining the Gaussian wave packet (the solutions of the Schrödinger equation) for the system.

## Materials and Methods

From the Hamiltonian of a free particle, the Hermitian linear invariant operator of the system will be constructed. Plane wave solution *φ*_*λ*_(*x*, *t*) of the eigenvalue equation of the invariant operator will be derived from a straightforward evaluation. From the theory of the Lewis-Riesenfeld^21^, one can confirm that the wave function *ψ*_*λ*_(*x*, *t*) of the system can be represented in terms of this plane wave solution, i.e., *ψ*_*λ*_(*x*, *t*) = *N*_0_*φ*_*λ*_(*x*, *t*) exp[*iα*_*λ*_(*t*)], where *N*_0_ is a normalization constant and *α*_*λ*_(*t*) is a time-dependent phase. With the aid of the Schrödinger equation, we will determine the analytical form of *α*_*λ*_(*t*). We then have the complete wave function *ψ*_*λ*_(*x*, *t*) which corresponds to the plane wave.

To obtain the Gaussian-type wave function Ψ(*x*, *t*), we will introduce a suitable weight function *g*(*λ*). Then, through the integration 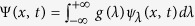, the Gaussian wave function Ψ(*x*, *t*) which satisfies the Schrödinger equation will be derived. Finally, quantum characteristics of the free particle will be analyzed by making use of Ψ(*x*, *t*).

## Results

### Invariant and the generalized coherent state

Let us consider a free particle that travels with a momentum *p*. To study the quantum properties of this system, it is necessary to find the solutions of the Schrödinger equation


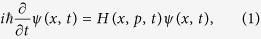


with Hamiltonian *H*(*x*, *p*, *t*) of the form


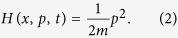


As mentioned in the introductory section, the customarily obtained solutions of Eq. (1) with Eq. (2) are delocalized ones. This delocalization leads to a continuous energy spectrum for the system; in this case, the construction of the coherent state is not an easy task. To overcome this difficulty, we introduce an invariant operator and derive its eigenstate which is localized at a position. The eigenstate *φ*_*λ*_(*x*, *t*) obtained in this way is almost the same as a certain Schrödinger solution *ψ*_*λ*_(*x*, *t*). In fact, the difference between them is just a multiplication by a phase factor exp[*iα*_*λ*_(*t*)] (see Materials and Methods section). This leads to a localized Schrödinger solution (a generalized quantum solution) that corresponds to the coherent state.

We shall utilize the Lewis-Riesenfeld method^21^ in order to obtain the generalized quantum solutions for the time behavior of the system in the configuration space. To proceed our theory, it is necessary to find an invariant operator *I*(*t*) satisfying the identity


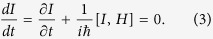


Clearly, the meaning of this equation is equivalent to saying that, if *φ*_*λ*_(*x*, *t*) is an eigenfunction of *I*(*t*) with a time-*in*dependent eigenvalue *λ*, we can find a solution of the Schrödinger equation in the form *ψ*_*λ*_(*x*, *t*) = exp[*iα*_*λ*_(*t*)]*φ*_*λ*_(*x*, *t*), where *α*_*λ*_(*t*) satisfies the eigenvalue equation for the Schrödinger operator:


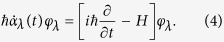


Following ref. 13, we suppose that the solution of Eq. (3) takes the form


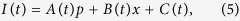


where *A*(*t*), *B*(*t*), and *C*(*t*) are time-dependent coefficients that will be determined. From the substitution of Eqs. (2) and (5) in Eq. (3), we have the time-dependent coefficients such that


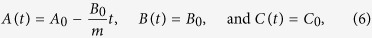


where *A*_0_, *B*_0_, and *C*_0_ are arbitrary real constants.

The eigenstates *φ*_*λ*_(*x*, *t*) of *I*(*t*) are the solutions of the equation


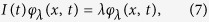


where the corresponding eigenvalues *λ* are time-*in*dependent. We can show that the solutions *φ*_*λ*_(*x*, *t*) of Eq. (7) are of the form


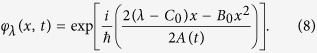


Substituting Eq. (8) into Eq. (4) and executing the integration, we obtain


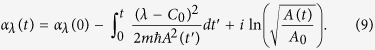


Here, the first relation in Eq. (6) is used. Notice that the logarithmic term in the above equation decreases as the time increases. We now confirm that the time-dependent normalized wave functions *ψ*_*λ*_(*x*, *t*) can be written as


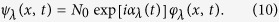


From the normalization condition for the Dirac delta function, 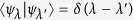, we have the normalization factor as 
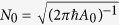
. Therefore the physical orthogonal wave functions *ψ*_*λ*_(*x*, *t*), which are solutions of the Schrödinger equation (1), are given by


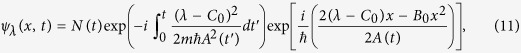


where we have taken *α*_*λ*_(0) = 0 and the new normalization factor is given by 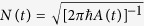.

Furthermore, the general wave function Ψ(*x*, *t*) is then written as


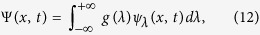


where *g*(*λ*) is a weight function which determines the state of the system in a way that Ψ(*x*, *t*) becomes square integrable, i.e., that the integration 
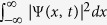
 becomes a time-*in*dependent finite constant. Any suitable choice of *g*(*λ*) yields a conventional solution as the Gaussian wave-packet function. Let us now choose the weight function as a Gaussian form too:


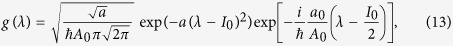


where *a*, *a*_0_, and *I*_0_ are positive real constants.

Substituting Eqs. (11) and (13) into Eq. (12) and accomplishing the integration by changing the integration variable without loss of generality as *λ* → *λ* + *I*_0_, we obtain the normalized Gaussian wave function in the form


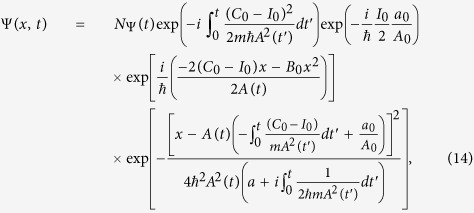


where 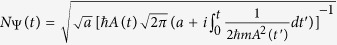. We can establish a natural relation between the classical and quantum descriptions of free particles by taking advantages of the characteristics of this wave function. For the case *B*_0_ = 0 as a particular example, the time-dependent invariant reduces to a simple one that is linear only in *p* while it has nothing to do with *x*. If we further impose *I*_0_ = 0. our wave function given in Eq. (14) reduces to those of the well-known “localized states” that describe a free particle as the product of a plane wave and a Gaussian state that has been derived in the most textbooks of quantum mechanics (see for example ref. 23).

### Quantum analysis of the free particle system

We now evaluate the mean value of *x* and *p* in the state Ψ(*x*, *t*). To do this, we put *I*_0_ = *a*_0_*B*_0_ for convenience. By executing a minor mathematical procedure using the relation 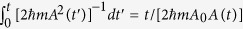, we find that


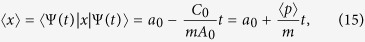


which is nothing but the classical position *x*_*c*_(*t*). By a similar method, we also have


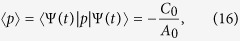


which is a classical momentum *p*_*c*_(*t*). The quantum mechanical expectation values for position and momentum, determined with the Gaussian wave function, satisfy the classical equations of motion for a free particle.

Further, we can derive the position uncertainty from 
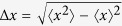
. A basic evaluation for this results in


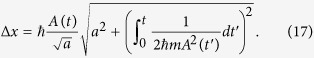


Meanwhile, the momentum uncertainty yields


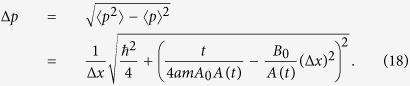


**Figure 1:**
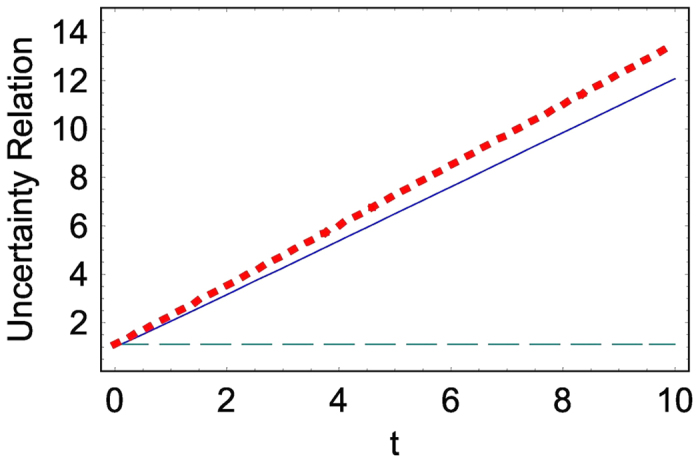
Time evolution of the uncertainty relation, Δ*x*Δ*p* (thick dotted line). We also represented Δ*x* (solid line) and Δ*p* (dashed line). We have used *a* = 1, *A*_0_ = 1, *B*_0_ = −1, *m* = 1, and *ħ* = 1.

The multiplication of Eq. (17) with Eq. (18) leads to the uncertainty relation:


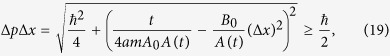


which is always larger than *ħ*/2 that is the minimally acceptable uncertainty in quantum mechanics. From Fig. 1, we see that Δ*x* increases with time while Δ*p* almost does not vary. Hence, the corresponding uncertainty relation increases with time. Such an increasing property of the uncertainty relation for the free particle coincides with the analysis of the uncertainty relation associated with the wave packet developed in ref. 24 (see Eq. (2.20) in that reference). This consequence has been originated from the spreading of the wave packet and is interesting if we think that the uncertainty relation for the simple harmonic oscillator does not vary with time and is always kept *ħ*/2 in the coherent state.

Now we can rewrite Eq. (14) in terms of 〈*x*〉 and 〈*p*〉 as


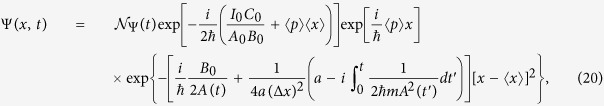


where 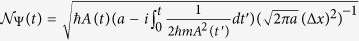. Moreover, the time-dependent probability density associated with this wave packet is Gaussian for all times and is given by


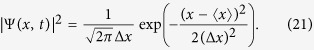


**Figure 2:**
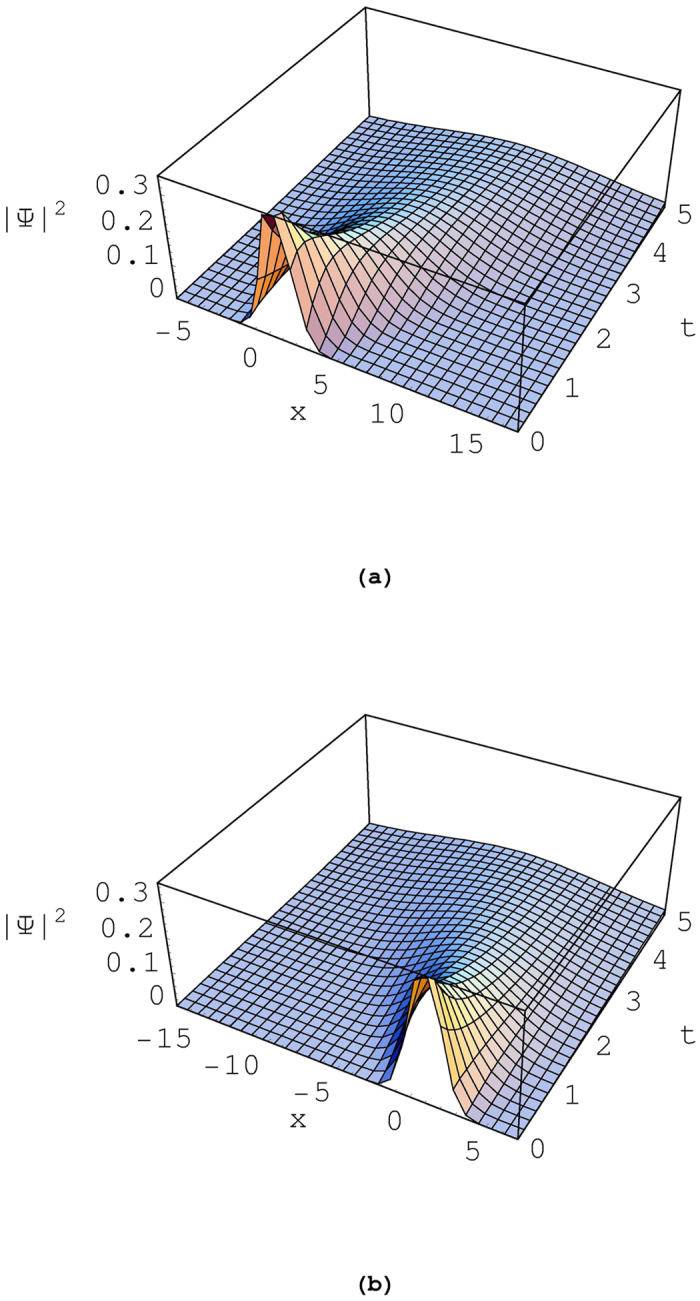
Time evolution of the wave packet, |Ψ(*x*, *t*)|^2^. We have used *a* = 1, *A*_0_ = 1, *B*_0_ = −1, *m* = 1, *ħ* = 1, and *a*_0_ = 1. The value of *C*_0_ is −1 for (**a**) and 1 for (**b**).

We see from this equation that the width of the wave packet at any time *t* is identical to Δ*x*. This wave packet is illustrated in Fig. 2. Figure 2(a) corresponds to the wave packet for a particle moving along the positive *x* direction, while 2(b) for a particle moving along the negative *x* direction. The curve is peaked at 〈*x*〉 and has a sharp-fall on either side. It is also readily verified that the time-dependent probability density is conserved: 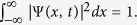


From Eq. (21), we can confirm that the wave described by Eq. (20) is a Gaussian wave packet centered at 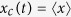. The width Δ*x*(*t*) of this Gaussian wave varies with time. So, during time *t*, the center of the packet travels from *x*_*c*_ = 0 to 
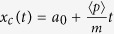
 while its width expands from 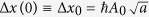 to 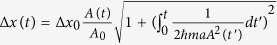. Although the shape of the wave packet is always kept to be Gaussian, the packet undergoes a distortion through its spreading. As a consequence, the width of the packet gradually becomes broader over time whereas its height, 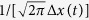, decreases.

For further analysis of the packet, let us define a displacement unitary operator *D*(*α*, *t*) as


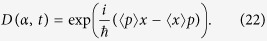


It is interesting that the displaced Gaussian wave function Ψ(*x*, *t*) given in Eq. (20) can be regarded as a refined version of the standard coherent state obtainable by applying the displacement operator, *D*(*α*, *t*), to the ground state Ψ_0_(*x*, *t*), i.e.,


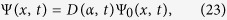


where Ψ_0_(*x*, *t*) corresponds to a particle at rest 〈*p*〉 = 0 at the origin 〈*x*〉 = 0:


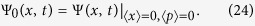


It is easy to notice that, Ψ_0_(*x*, *t*) is obtained by replacing 〈*x*〉 and 〈*p*〉 with 〈*x*〉 = 0, and 〈*p*〉 = 0 in the wave function given in Eq. (20).

If we think the fact that the mean values of the canonical variables correspond to their classical counterpart ones in coherent state, i.e., 〈*x*〉 = *x*_*c*_ and 〈*p*〉 = *p*_*c*_, the action of *D*(*α*, *t*) on a wave function in the *x*-representation, gives 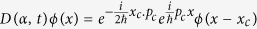. In addition, the coordinate and momentum operators can be changed by the displacement operator to be


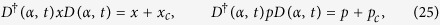


as expected. Indeed, the coherent state developed here plays an exquisite role for connecting quantum descriptions of the free particle with the counterpart classical descriptions.

## Discussion

In this work, we have reconsidered the linear invariant proposed by Bagrov *et al*.^13^ in order to derive quantum solution of the motion of the free particle. We have shown that, if we take this linear invariant operator to be an Hermitian one, then a coherent state solution can be naturally derived based on the Lewis-Riesenfeld approach^21^.

We see from Eq. (23) that *D*(*α*, *t*) given in Eq. (22) transforms a wave function Ψ_0_(*x*, *t*) corresponding to a particle at rest, 〈*p*〉 = 0, at the origin, 〈*x*〉 = 0, into a wave function Eq. (20) associated with the particle passing a position 〈*x*〉 with a momentum 〈*p*〉. We therefore see that the Gaussian state Ψ(*x*, *t*) [Eq. (20)] is created from the ground state Ψ_0_(*x*, *t*) by a unitary operator *D*(*α*, *t*). This state, constructed in the Glauber manner by acting the displacement operator on the vacuum state Ψ_0_(*x*, *t*) (defined by 〈*p*〉 = 0 and 〈*x*〉 = 0), is the coherent state of a free particle. The quantum expectation values for position and momentum, determined in the coherent state, satisfy the classical equations of motion for a free particle. Furthermore, as we have already seen, the coherent state remains a coherent one during its evolution with time. That is why the coherent states are used to study the classical limit of quantum mechanics. This coherent state has a “center of mass” moving along the trajectory of the corresponding classical particle. The trajectory is determined by the classical initial position and initial momentum. From these analyses, we can confirm that there is a complete quantum-classical correspondence for the free particle.

## Additional Information

**How to cite this article**: Maamache, M. *et al*. Analyzing generalized coherent states for a free particle. *Sci. Rep*. **6**, 30538; doi: 10.1038/srep30538 (2016).
